# HIF-1 and tumour radiosensitivity

**DOI:** 10.1038/sj.bjc.6603201

**Published:** 2006-05-30

**Authors:** B J Moeller, M W Dewhirst

**Affiliations:** 1 Department of Radiation Oncology, Duke University Medical Center, Box 3455, Room 201 MSRB, Research Drive, Durham, NC 27710, UK

**Keywords:** HIF-1, tumour, vessel, radiosensitivity

## Abstract

Hypoxia-inducible factor-1 (HIF-1) plays important roles in regulating radiosensitivity, making it a potentially promising target for tumour radiosensitisation. Here, we discuss the rationale for, and the potential pitfalls of, combining HIF-1 blockade with radiotherapy. In doing so, we describe clinical scenarios in which HIF-1 inhibition might optimise tumour radiosensitivity.

Hypoxia is an important contributor to tumour radioresistance (Brizel *et al*, 1997). Oxygen increases the cytotoxicity of radiation, resulting in roughly a three-fold difference in radiosensitivity between hypoxic and aerobic cells. This phenomenon, termed the oxygen effect, is widely attributed to oxygen's ability to chemically modify radiation-induced DNA damage, creating adducts that are not easily repaired by cells (Roots and Smith, 1974).

As we have come to better understand how tumours respond and adapt to hypoxia, it has become apparent that there may also be biological mechanisms that contribute to the oxygen effect. Owing to an imbalance in oxygen supply and demand, most solid tumours are hypoxic (Dewhirst, 2003). As a result, signaling pathways stimulated by hypoxia are commonly activated in tumours (Harris, 2002). Arguably, the best understood of these pathways is controlled by hypoxia-inducible factor-1 (HIF-1). Hypoxia-inducible factor-1 is a transcription factor activated by hypoxia that modulates more than 100 genes involved in regulating important processes such as metabolism, proliferation, apoptosis, and angiogenesis (Semenza, 2003).

Teleologically, the downstream effects of HIF-1 serve to help the cell adapt to hypoxic stress. In doing so, they change the tumour phenotype in ways that might also impact radiosensitivity; some positively, and some negatively (Table 1). As some of these HIF-1-mediated processes will be more predominant in certain tumours, it is likely that HIF-1 may have varying effects on radiosensitivity from tumour to tumour. In fact, there are some clinical data, which support this concept. In oropharyngeal cancer, high levels of HIF-1 expression have been shown to predict for poor local control in advanced disease (Aebersold *et al*, 2001), while predicting for superior local control in early-stage disease (Fillies *et al*, 2005).

The above data suggest that HIF-1 influences tumour radiosensitivity, but that the degree and direction of that influence may be dependent on context. Here, we hope to begin building an understanding for how the tumour phenotype affects the relationship between HIF-1 and tumour radiosensitivity. We will attempt to extrapolate from this how HIF-1 blockade might best be used to capitalise on its potential for tumour radiosensitisation.

## HOW RADIATION AFFECTS HIF-1

As shown by the clinical data mentioned above, pretreatment HIF-1 expression levels influence local control for irradiated tumours. This raises the question of how HIF-1 activity varies during and after radiotherapy, as these are the times when the protein should function as a modulator of radioresistance. As it turns out, radiation causes HIF-1 expression levels to increase in tumours (Moeller *et al*, 2004). The effect is dose-responsive, but does not appear to depend on dose fractionation. The upregulation begins as early as 24 h after treatment, and has been shown to endure for as long as 1 week. Coincident with this effect, several important downstream targets of HIF-1 are also upregulated in irradiated tumours. These proteins, including vascular endothelial growth factor (VEGF), plasminogen activator inhibitor-1, and carbonic anhydrase IX, serve as the longer-lived mediators of the radiation-induced HIF-1 effect.

The mechanisms behind this effect are interesting and, importantly, reveal nuances that may be clinically relevant. Two pathways have been worked out to explain how irradiation leads to HIF-1 activation (Moeller *et al*, 2004). The two share a common origin: radiation-induced tumour reoxygenation. As mentioned above, radiation preferentially kills well-oxygenated and highly metabolic tumour cells. The death of these cells frees up oxygen to distribute to regions of the irradiated tumour that were previously hypoxic. Tumours are also debulked by the cell death occurring after radiation, freeing up space for vessels to expand and improve blood flow and nutrient delivery to starved regions of tumour tissue. The end result is that oxygen levels in tumour tissue tend to be higher after irradiation, an effect termed ‘reoxygenation’.

Paradoxically, increasing tumour oxygenation in this way causes activation of the HIF-1 pathway, which is normally responsive to decreased oxygenation. Part of the explanation for this comes from the oxidative stress generated during tumour reoxygenation. After irradiation, during reoxygenation, free radical species accumulate in tumour tissue and lead to overexpression of HIF-1 (Moeller *et al*, 2004). It is unknown precisely through what mechanism this occurs, but several theories have been put forward. One postulates that free radical species generated in the mitochondria during hypoxia are the signal used by the cell to sense oxygen deprivation and that they are, therefore, capable themselves of mimicking hypoxia (Chandel *et al*, 2000). Another suggests that reactive nitrogen species inhibit the cellular machinery that breaks down HIF-1 during normoxia, causing HIF-1 to function as though the cells were hypoxic (Metzen *et al*, 2003). But whatever the cause, reoxygenation clearly changes the redox environment in irradiated tumours, causing accumulation of HIF-1.

Apart from setting off this oxidative pathway, reoxygenation also affects the translational machinery in the irradiated tumour cell, further contributing to the radiation-induced activation of the HIF-1 pathway. When a cell becomes stressed, it strives to conserve energy by, in part, slowing down protein synthesis. This is accomplished through a variety of mechanisms (Wouters *et al*, 2005). One involves dynamic regulation of protein translation through so-called stress granules. Stress granules are cytosolic polymers made up of mRNA, ribosomal subunits, and various other proteins (Kedersha *et al*, 1999). They serve as points of triage during stress, differentiating which transcripts need urgent translation and which can be sequestered in the granule for translation at a more favourable point in time (Kedersha and Anderson, 2002). As long as the cell has not been irreversibly damaged, the granules depolymerise once the stress is alleviated, and the once-sequestered transcripts go on to be translated normally.

Stress granules play a role in activating the HIF-1 pathway after tumours are irradiated (Moeller *et al*, 2004). They form in response to hypoxia and bind, among other things, mRNA transcribed off of HIF-1-regulated genes. This effect is not absolute, as proteins downstream of HIF-1 are synthesised during hypoxia. However, the magnitude of their upregulation is blunted by the activity of the stress granules. As proof of this, when reoxygenation causes the stress granules to disassemble, new protein translation causes synthesis of downstream HIF-1 targets to increase approximately two-fold. This provides the second mechanism through which radiation upregulates HIF-1 activity in tumours.

In summary, radiation causes upregulation of the HIF-1 pathway in tumours through two mechanisms dependent on reoxygenation. The resulting increased levels of free radical species within the tumour caused the HIF-1 protein to accumulate. At the same time, stress granules are depolymerised, reversing a partial blockade on protein synthesis in the HIF-1 pathway. Together, these effects significantly upregulate the HIF-1 pathway in the irradiated tumour.

## HOW HIF-1 AFFECTS TUMOUR RADIATION RESPONSE

As discussed above, data exists to suggest that HIF-1 plays a role in determining tumour radiosensitivity. Until recently, no study had been carried out to examine the mechanisms behind this relationship. These details are important to understand as they may lend further insight into why HIF-1 may differentially radiosensitise certain tumours. Moreover, this knowledge may reveal which tumours might be maximally radiosensitised by HIF-1 blockade.

To date, the effect HIF-1 has on tumour radiosensitivity has been ascribed to four different processes: its impact on apoptosis, metabolism, proliferation, and the tumour vasculature. It is not yet known whether any of these contributes more or less than the others to the overall effect so, for now, each should be considered as important as the next. We will examine each, in turn, below.

In general, HIF-1 has a complicated relationship with apoptosis. As mentioned above, HIF-1 has been shown in different situations to be both proapoptotic and antiapoptotic. In the irradiated cell, however, HIF-1 appears to have a net proapoptotic effect. Normally, ionising radiation induces breaks in DNA that are sensed by the cell, setting off a cascade of molecular reactions that help determine which of the many possible fates (i.e. repair, apoptosis, mitotic death) the cell should meet. For cells that eventually undergo apoptosis, one of the most important events in this cascade is the activation of p53.

HIF-1 exerts its effect on radiation-induced apoptosis, at least in part, through interacting with p53. Tumour cells exposed to both hypoxia and radiation undergo apoptosis through a p53- and HIF-1-dependent mechanism (Moeller *et al*, 2005b). HIF-1 enhances phosphorylation of p53 in the irradiated cell, promoting caspase cleavage and, eventually, apoptosis. Of note, HIF-1 appears to have no impact on radiation-induced apoptosis in the p53-null human prostate cell line, PC-3. When p53 is reintroduced to the PC-3 line, it regains HIF-1-dependent sensitivity to radiation-induced apoptosis. It remains to be seen whether the link between HIF-1 and radiation-induced apoptosis is as closely tied to p53 in other cell lines. There is ample reason to believe it may not be, as HIF-1 is known to affect several p53-independent apoptotic mechanisms, such as BNIP3 (Bruick, 2000).

HIF-1 can also affect tumour cell clonogenicity following irradiation by altering cellular metabolism. It governs the expression of a host of proteins involved in glycolysis, and serves an important role in maintaining energy levels during hypoxia. Consequently, tumours deficient in HIF-1 have difficulty maintaining ATP levels (Griffiths *et al*, 2002; Moeller *et al*, 2005b) – a state which might increase clonogenicity after irradiation (Luk and Sutherland, 1987). Indeed, if tumour cells are made to be hypoxic in a glucose-limiting environment, without the aid of HIF-1's glycolysis-promoting effects, their metabolic rates drop, ATP levels fall, and *in vitro* clonogenicity increases (Moeller *et al*, 2005b).

HIF-1 also affects tumour cell clonogenicity by altering the kinetics of cellular proliferation. This effect is highly dependent on the local microenvironment and, therefore, varies considerably from place to place within a tumour. In regions where both hypoxia and glucose are limited, HIF-1 maintains tumour proliferation, likely by supporting bioenergetics. In regions where hypoxia alone is limiting, HIF-1 promotes cell cycle blockade, likely by modulating p21 and p27 (Goda *et al*, 2003). The overall impact of these combined effects is that HIF-1 radiosensitises tumours by keeping cells proliferative in nutrient-depleted regions of the tumour tissue (Moeller *et al*, 2005b).

The final known mechanism linking HIF-1 activity to tumour radiosensitivity involves the regulation of vascular radiosensitivity. The degree to which the tumour vasculature is damaged by radiation has been shown to be an important determinant of overall responsiveness of tumours to ionising radiation in animal models (Garcia-Barros *et al*, 2003). The radiosensitivity of endothelial cells within a tumour, in turn, appears to be highly dependent on their exposure to proangiogenic stimulants. Proangiogenic cytokines, such as VEGF and basic fibroblast growth factor, have been found to induce significant radioprotection in endothelial cells (Gorski *et al*, 1999; Paris *et al*, 2001).

HIF-1, which promotes the expression of a variety of proangiogenic cytokines, is poised to be a major regulator of this behavior in tumours. Conditioned media taken from wild-type tumour cells is much more efficient at inducing resistance to radiation-induced death in endothelial cells than is that taken from HIF-1-deficient tumour cells (Moeller *et al*, 2004). In line with this finding, the vasculature of HIF-1-deficient tumours undergoes significantly more regression following irradiation than does the vasculature of their wild-type controls (Moeller *et al*, 2005b). Through its protective effects on the surrounding vascular endothelium, then, HIF-1 serves as a powerful radioprotective factor for tumours.

**Figure 1:**
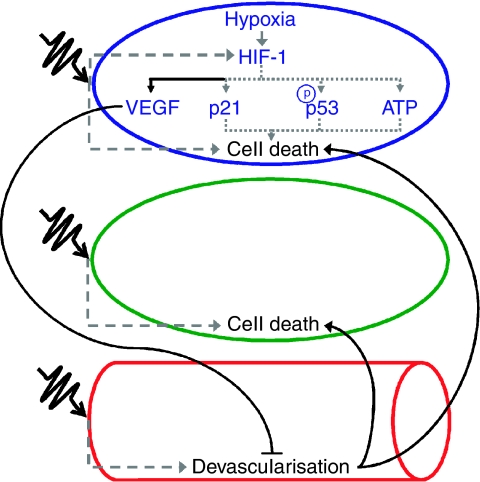
A representation of HIF-1's effects on radiosensitivity for a HIF-1-expressing tumour cell (blue), a non-HIF-1-expressing tumour cell (green), and a tumour vessel (red). Dashed arrows depict the normal effects of ionising radiation. Dotted arrows show HIF-1-mediated pathways that sensitise tumours to radiation. Solid arrows show HIF-1-mediated pathways that protect tumours from radiation, whose blockade might radiosensitise tumours. ‘VEGF’ symbolises the proangiogenic pathway, ‘p21’ the mitotic pathway, ‘p-p53’ the apoptotic pathway, and ‘ATP’ the metabolic pathway. Note that in this schematic, non-HIF-1-expressing cells are still radiosensitised by HIF-1 blockade.

The above data show that HIF-1 has divergent effects on radiosensitivity: sensitisation of tumour cells and protection of stromal endothelial cells (Figure 1). It is critical to understand how these divergent mechanisms come together to bring about a change in tumour radiosensitivity. If the net result of HIF-1 activity is to promote radioresistance in tumours, HIF-1 blockade would seem to be a promising strategy for tumour radiosensitisation. However, three of the four HIF-1-mediated effects described – enhancing apoptosis, metabolism, and proliferation – result in tumour radiosensitisation, while only one – vascular protection – promotes tumour radioresistance. One might predict from this, then, that HIF-1 blockade could interfere with radiotherapy. Studies have shown, however, that HIF-1-deficient tumours are more radiosensitive than their wild-type counterparts (Zhang *et al*, 2004; Moeller *et al*, 2005b; Williams *et al*, 2005).

How can this apparent discrepancy be explained? One possibility is that HIF-1 promotes tumour radioresistance through some other effects, not yet realised or described. Another is that the vascular radiosensitisation caused by HIF-1 blockade is a more powerful influence on tumour radiosensitivity than are the other factors discussed. There are some data to support these hypotheses. It has been shown in one experimental system that eliminating the hypoxic fraction of a HIF-1-deficient tumour immediately prior to irradiation does not alter the tumour regrowth rate after treatment (Williams *et al*, 2005). What this experiment suggests is that, in contrast to wild-type tumours, the hypoxic fraction of HIF-1-deficient tumours does not contribute to tumour regrowth after irradiation. One might surmise from this that even though HIF-1 blockade causes some degree of radioresistance in the hypoxic cell, the combination of hypoxia and HIF-1 inhibition render that cell, overall, nonclonogenic after irradiation. Moreover, since the radiosensitising effects of HIF-1 blockade occur at a distance – damaging all tumour cells fed by the affected vasculature – the positive effects apply more broadly over the tumour than do the negative effects. Whatever the explanation, these data suggest that HIF-1 blockade is a viable strategy for tumour radiosensitisation.

## CLINICAL CORRELATION

There are lessons to be learned from the mechanisms connecting radiation to HIF-1, and HIF-1 to radiosensitivity, which may be relevant for the use of HIF-1 blockade as a tumour radiosensitiser (Table 2). First, the degree of tumour hypoxia is likely to play a role in determining how HIF-1 blockade influences tumour radiosensitivity. Of course, there are numerous hypoxia-independent stimuli for HIF-1 activation (Dery *et al*, 2005). As a result, tumours that are well-oxygenated yet signal robustly through PI3K, for example, may have sufficient HIF-1 activity to influence their radiosensitivity. However, tumours need to be hypoxic to undergo radiation-induced HIF-1 activation since reoxygenation is required to initiate this pathway. Therefore, both baseline HIF-1 expression levels and oxygenation are likely important determinants of how tumour radiosensitivity will respond to HIF-1 blockade. Second, since stress granules do not depolymerise after a cell experiences a lethal threat, tumours need to be made up of mostly viable tissue in order to maximally activate the HIF-1 pathway after radiation. Therefore, tumours with low HIF-1 activity levels, little or no hypoxia, or tumours with vast amounts of necrosis are unlikely to undergo much HIF-1 activation in response to radiotherapy. Consequently, HIF-1 blockade is less likely to add benefit to radiation in such tumours. Third, since HIF-1 collaborates with p53 to promote apoptosis, scheduling HIF-1 inhibition with radiation may be a challenge in p53-positive tumours.

Considerations such as these may influence how HIF-1 blockade is used in the clinic. This is particularly relevant now, as there are several agents currently being developed as potential HIF-1 inhibitors for use in oncology. One frontrunner is topotecan, a topoisomerase I inhibitor which blocks HIF-1 translation at doses lower than those required for the drug to damage DNA (Rapisarda *et al*, 2004). Topotecan is currently undergoing a phase one clinical study as a HIF-1 inhibitor. A second promising class of HIF-1-inhibiting agents undergoing active clinical development are the analogues of 2-methoxyestradiol from EntreMed, Inc, which downregulate HIF-1 at the post-translational level (Mabjeesh *et al*, 2003). A large number of other ‘hits’ from various small molecule screens have also been presented as potential clinical HIF-1 inhibitors. Only time will tell which of these, if any, are efficacious in human tumours and augment the effects of standard cytotoxic therapies such as radiation.

Another potential strategy to counter the radioprotective effects of HIF-1 would be to inhibit the mechanisms by which radiation causes HIF-1 activation. This has been done in a preclinical model using a manganese porphyrin compound – a mimetic of superoxide dismutase – to scavenge the free radical species generated during radiation-induced reoxygenation (Moeller *et al*, 2004). Combining these agents with radiotherapy delays tumour regrowth significantly over that seen with either treatment alone (Moeller *et al*, 2005a). As the manganese porphyrin compounds also protect normal tissues from radiation damage, this may be a powerful clinical therapeutic option.

Finally, it is worth mentioning here that there are no data yet on whether HIF-1 blockade might impact normal tissue radiosensitivity. As HIF-1 is not typically active in most healthy normal tissues, one would expect that its inhibition would not affect normal tissue radiosensitivity. There may, however, be exceptions to this rule. There are some normal tissues, such as the liver and the thymus, that are hypoxic at baseline (Arteel *et al*, 1995; Hale *et al*, 2002). HIF-1 may play a homeostatic role in such organs, potentially important in the recovery from radiation damage. Indeed, HIF-1 has been implicated in the response to injury and inflammation in normal tissues (Maeno *et al*, 2005), processes which have much in common with the response to radiation damage. It remains an important yet unresolved issue to determine whether inhibiting the function of HIF-1 in normal tissues would impair their ability to heal after being irradiated.

## Figures and Tables

**Table 1:** HIF-1 modulates many processes, which might cause tumours to be more or less radiosensitive

**HIF-1-mediated effect**	**Potential impact on radiosensitivity**
Cell cycle arrest (Goda *et al*, 2003)	Decreased
Proapoptotic (Carmeliet *et al*, 1998)	Increased
Antiapoptotic (Piret *et al*, 2004)	Decreased
Enhanced glycolysis (Semenza *et al*, 1994)	Increased
Angiogenesis (Carmeliet *et al*, 1998)	Decreased (Gorski *et al*, 1999)

**Table 2:** Characteristics of tumours that might conceivably limit the influence of HIF-1 on radiosensitivity and/or the efficacy of combined HIF-1 blockade and radiotherapy

**Tumour characteristic**	**Potential effect**	**Potential consequence**	**Therapeutic implication**
Scant hypoxia	Limited reoxygenation after irradiation	Limited HIF-1 activation after irradiation	Limited additional benefit of HIF-1 blockade
Abundant necrosis	Stress granules fail to depolymerize	Limited HIF-1 activation after irradiation	Limited additional benefit of HIF-1 blockade
p53 Positive	HIF-1/p53 interaction promotes apoptosis	Enhanced radiation-induced apoptotic cell death	HIF-1 blockade may decrease apoptotic cell death
Poor anatomic location	—	HIF-1 blockade may radiosensitize some normal tissues	HIF-1 blockade may fail to widen the therapeutic ratio
